# The stromal microenvironment and ovarian aging: mechanisms and therapeutic opportunities

**DOI:** 10.1186/s13048-023-01300-4

**Published:** 2023-12-13

**Authors:** Lu Shen, Junfeng Liu, Aiyue Luo, Shixuan Wang

**Affiliations:** 1 National Clinical Research Center for Obstetrical and Gynecological Diseases, Department of Obstetrics and Gynecology, Tongji Hospital, Tongji Medical College, Huazhong University of Science and Technology, Wuhan, 430030 China; 2 Tongji Medical College, Huazhong University of Science and Technology, Wuhan, 430030 China

**Keywords:** Aging, Ovary, Stroma, Microenvironment

## Abstract

For decades, most studies of ovarian aging have focused on its functional units, known as follicles, which include oocytes and granulosa cells. However, in the ovarian stroma, there are a variety of somatic components that bridge the gap between general aging and ovarian senescence. Physiologically, general cell types, microvascular structures, extracellular matrix, and intercellular molecules affect folliculogenesis and corpus luteum physiology alongside the ovarian cycle. As a result of damage caused by age-related metabolite accumulation and external insults, the microenvironment of stromal cells is progressively remodeled, thus inevitably perturbing ovarian physiology. With the established platforms for follicle cryopreservation and in vitro maturation and the development of organoid research, it is desirable to develop strategies to improve the microenvironment of the follicle by targeting the perifollicular environment. In this review, we summarize the role of stromal components in ovarian aging, describing their age-related alterations and associated effects. Moreover, we list some potential techniques that may mitigate ovarian aging based on their effect on the stromal microenvironment.

## Introduction

With improved social and economic status in modern life, women are inclined to delay family planning and childbearing, which has led to an increased need for strategies to preserve fertility and delay reproductive aging. However, female reproduction sharply declines with chronological aging. The live birth rate drops from 26% at age 35 to 1% at age 42, showing a robust linear decrease (10% per year). This means that women still face the inevitable dilemma of subfertility after middle age [1]. Additionally, women suffer an increased risk of age-related diseases after menopause, including cardiovascular disease, osteoporosis, Alzheimer’s disease, and diabetes, among others, suggesting that age-associated ovarian dysfunction is a pacemaker of general organic aging [2–4]. As the human lifespan is steadily being prolonged and women are becoming increasingly concerned about healthy aging, researchers must determine the mechanisms of ovarian aging and identify potent therapeutic strategies for its postponement.

The natural aging of ovary is closely associated with a decline in reproduction and abnormal endocrine function that manifests as infertility, irregular menstruation. The ovarian follicle is the unit core executing the two fundamental functions of ovary, i.e., endocrine and fertility. Ovarian aging is characterized by progressively declining quantity and compromised quality of follicles. The exhaustion of ovarian reserve has been reported to subvert folliculogenesis. In response to the decreased growing follicle reserve, circulating FSH levels increase to promote the recruitment of primordial follicles and to rescue more selectable antral follicles from atresia. This has been viewed as a compensatory mechanism that acts at the expense of follicle quality [5, 6]. With the activated primordial follicle activation (PFA), phosphatidylinositol 3-kinase (PI3K)/AKT (PI3K/AKT) pathway mediates DNA damage and impairs the repair capacity of oocytes through ribosomal protein S6 (rpS6) [7–10]. Second, primary follicles support each other for growth through paracrine signaling, but the decreased follicle density leads to the compromised supportive capability and growth [11, 12]. Moreover, according to the production-line hypothesis, optimal follicles are recruited first leaving the poorer follicles in the aged ovary to maximize the utilization of the best gametes [13]. On the other hand, the oocytes in follicles are decaying with a defective nuclear genome and/or cytoplasm during the long-term dormancy [14]. Shortened telomeres lead to the dysfunctional spindles, decreased chiasmata, and abnormal synapsis, etc. [15]. Therefore, oogonia recruited at a late age may harbor the shortened telomeres because of replicative senescence, as they are the last batches exiting the oogonial cell cycle based on the productive-line theory mentioned above [16]. The balance between spontaneous mutation and repair in the nucleus is progressively tilted, leading to accumulated DNA damage and chromosomal instability. Organelles in immature oocytes maintain a low rate of metabolism, even though they are quiescent, as chronic toxicity is provided by metabolites [17]. For example, mitochondria start to produce excessive amounts of oxidants. This leads to mitochondrial DNA (mtDNA) mutations, which exacerbate energy insufficiency [18, 19]. Additionally, the defective crosstalk between oocytes and granulosa cells such as reduced transzonal projections (TZPs) gap junctional coupling, and oocyte-derived microvilli (Oo-Mvi), also leads to the loss of oocyte quality [20, 21]. Notwithstanding, the factors leading to ovarian aging and their mechanisms of actions remain unexplored.

**Fig. 1:**
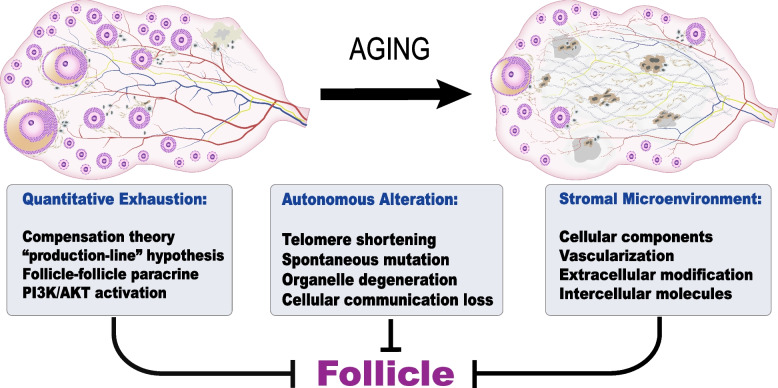
Age-related changes in the intrinsic characteristics and the extrinsic microenvironment of follicles

Ovarian microenvironment plays a significant role in mediating somatic aging and follicular part. Orthotopic transplantation of ovarian tissue between young and aged mice demonstrated that a healthy stromal microenvironment plays an essential role in folliculogenesis [22]. In ovaries, follicles are surrounded by a variety of stromal cell types and microstructures, i.e., theca-interstitial cells, immune cells, nerve and blood vessels, and extracellular matrix (ECM) [23]. These components support follicles physically by providing the biological scaffold, and chemically by the paracrine effect of nutritional and signaling molecules. For example, early-stage follicles acquire their oxygen and blood supply from adjacent stromal microvasculature, but spindle abnormalities occur with reduced vascularization [24]. Macrophages are reported to be dispensable for folliculogenesis, and their ablation causes ovarian hemorrhage and disrupted steroidogenesis [25, 26]. Moreover, the deposition and resolution of ECM molecules determine the stiffness of the perifollicular environment, affecting the activation of primordial follicles [27]. Additionally, microenvironmental molecules associated with aging, i.e., advanced glycation end products (AGEs), reactive oxidative species (ROS), and inflammatory cytokines, are produced and accumulate in the perifollicular environment. They interact with stromal cells to affect follicle development synergistically [28]. In the context of somatic aging, the dysfunction of these components has been implicated in multiple pathophysiological changes, such as tissue fibrosis, inflamm-aging, and immune senescence. However, their manifestations and effects on ovarian aging have not yet been clarified. Therefore, in this review, we focus on the ovarian components surrounding follicles that mediate the crosstalk between the follicle and microenvironment. We discuss their age-associated alterations and effects. Moreover, we propose potent strategies that could rescue or alleviate the effect of aging on ovaries based on the techniques related with the stromal microenvironment (Fig. 1).

## Age-related changes in the perifollicular microenvironment

### Stroma

**Fig. 2:**
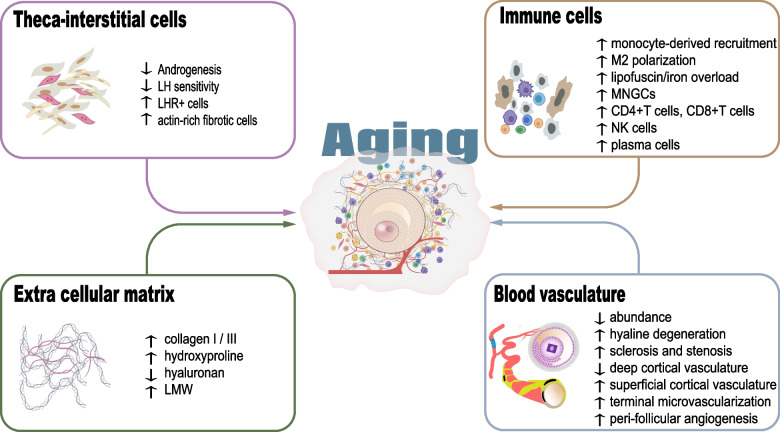
Evidenced age-related alterations of ovarian stromal components

Kinnear et al. characterized the cell types of the ovarian stroma into mainly two categories: 1) general cell types composing immune system, nerves, blood, and lymphatic system, etc.; and 2) ovary-specific cell types, including surface epithelium, tunica albuginea, rete ovarii, hilar cells, and most uncharacterized stromal cells [23]. The ovarian microenvironment comprises more than cells, as it also contains extracellular matrix molecules, secretory or/and soluble factors, metabolic products, etc. Some of these components interact with each other playing critical roles in regulating follicle development and germline cell differentiation. Herein, we mainly focused on the stromal components that have been documented to be associated with ovarian aging and discuss their age-related alterations, and roles in the peri-follicular microenvironment leading to ovarian dysfunction (Fig. 2 and Table 1).

**Table 1:** The physiological function and their age-related mechanisms of main stromal components in ovary aging

**Physiological Functions**	**Age-Related Alterations**	**Mechanisms in Ovarian Aging**
**Theca-interstitial cells**		
Architectural support [29] Paracrine [30, 31] Androgen production [32]	Androgen production ↓ [33] Phasic sensitivity of LH ↓ [34] PPARα expression ↓, DHEA synthesis ↓ [35] LH receptor expression ↑ Actin-rich fibrotic cells ↑ [36, 37]	Steroidogenesis ↓ [33] Oocyte quality ↓, apoptosis ↑ [35] Ovulatory dysfunction [38] Stromal fibrosis ↑ [37] Secondary follicle development ↓ [37]
**Immune cells**		
**Monocyte/macrophage (Mφ)**		
Folliculogenesis: granulosa cell proliferation, vascular integrity [25] Ovulation: pro-inflammation, matrix breakdown [39] Luteal phase: vascularization, progesterone synthesis [40, 41] Follicle atresia/luteolysis: scavenging debris/apoptotic cells [42]	Percentage of populations ↓: resident Mφ ↓, monocyte derived Mφ ↑ [43] Polarization from M1 to M2 [44] Phagocytotic function ↓ Iron/lipofuscin overload ↑ Multinucleated giant cells (MNGCs) ↑ [42, 45]	Granulosa proliferation ↓ [25] Follicle growth ↓ [25] Steroidogenesis (E2, P4) ↓ [25, 41] Corpus luteal hemorrhage ↑ [25] Corpus luteum formation/lysis ↓ [41] Stromal waste/debris ↑ [46]
**Other immune cells**		
Phagocytosis [47] Antigen presentation [48] Paracrine/autocrine [47]	CD4+ T cells ↑ NK cells ↑ CD8+ T cells ↓ [43] Plasma cells ↑ Naive CD4+ T cells ↑ [49]	Abnormal immunity [50, 51] Luteal regression [40] Autoimmune reaction [48]
**Vasculature**		
**Pericytes**		
Follicular vascularization [52] Initiate luteal angiogenesis: Endothelial cell migration Capillary outgrowth Vessel stablization [53]	Migration ↓ [54] Apoptosis ↑ Detachment ↑ Coverage ↓ [55] Differentiation to fibroblasts [56]	Altered angiogenesis [52] Vascular instability Luteal hemorrhage [53] Fibrosis [56]
**Smooth muscular cells**		
Constituting arterioles and muscular venules [57]	Migration ↑ Proliferation ↑ Hypotrophy [57]	NA
**Endothelial cells**		
Constituting blood/lymphatic vessels, secreting NO in response to hypoxia [58] Interaction with perivascular cells [59] Angiocrine [60]	Apoptosis ↑, Regeneration ↓, eNOS-NO ↓ [58] Senescence ↑ [61] Interaction with pericyte ↓ [62] Migration↓, proliferation ↓ [63] Suboptimal angiocrine [64]	Perifollicular angiogenesis ↓ [52] Postovulatory vascularization ↓ [59]
**Blood vasculature**		
Supportive architecture Delivering/removing nutrients and metabolites [65]	Stromal blood flow ↓ [66–68] Superficial cortex (> 30 yr): density ↑ [69] Deep cortical stroma (> 40 yr): abundance ↓ [69] Hyaline degeneration, sclerosis, and stenosis [67]	Primordial follicle activation ↑ Earliest follicle development ↑ [69] Terminal micro-vascularization [69]
**Lymphatic vasculature**		
Extravascular fluids homeostasis Hormone recruitment Immune cell transport [65]	Capillary rarefaction; dilated; contractile ↓ Permeable ↑ [70]	Secondary follicle development ↓ [71] Follicular fluid accumulation [72]
**Extra cellular matrix**		
Sequesteriation Signaling [73, 74] Biomechanics [75]	Collagen (type I and III) ↑ Hydroxyproline ↑ [76] Hyaluronan (HA) ↓ [77] Low molecular weight hyaluronan (LMW) ↑ [78]	Primordial follicle activation ↓ [75] Oocyte dormancy ↑ [27] Meiosis/maturation ↓ [77, 78] Ovulation ↓ [79] Granulosa cell proliferation ↓ Steroidogenesis ↓ [80]

#### Theca-interstitial cells

According to Kinnear et al., the interstitial part of the ovary is heterogeneous and composed of different cell types. Among the interstitial cells, fibroblast-like cells produce ECM molecules, which are involved in peri-follicle scaffolding and biomechanics. Spindle-shaped cells produce steroids, mainly androgens, and participate in follicle modulation [23]. Hummitzsch et al. compared gene expression profiles between the interstitial stroma, peri-preantral follicular pre-theca, theca interna of antral follicles, and tunica albuginea of bovine ovaries, and found minimal differences between the pre-theca and interstitial stroma [66]. This finding is consistent with the notion that theca cells are derived from interstitial cells in the stroma. These two types of cells were studied in mice, and together, they were termed ‘theca-interstitial cells’ or ‘stromal cells’ [67]. However, Richards et al. proposed that theca cells might be derived from two resources. Only those from progenitors migrating from mesonephros become androgen-producing cells, and others from ovarian indigenous stroma cells produce fibroblasts and smooth muscle cells [81]. Regardless, both cell types secrete matrix-related factors, providing architectural support and integrity to the perifollicular microenvironment. In injury conditions (incision or ovulation), interstitial cells stimulate primordial follicle activation via nerve growth factor (NGF) in mice [68, 82]. Moreover, they control the perifollicular vascular system and regulate the blood supply of follicles by secreting members of the transforming growth factor beta (TGF-β) superfamily and cell adhesion molecules (CAMs) [69, 83].

Theca cells play an essential role in follicle growth, mainly via producing androgens; moreover, cell types in the ovarian hilum, the mesovarium and the interstitial stroma may also synthesize and secrete androgens in postmenopausal ovaries [70–72]. In humans, serum androgen levels in women decrease in a biphasic pattern with age, steeply dropping during age 25–45 but with no significant change after age 45 [84]. Umehara et al. found an increased number of luteinizing hormone receptor (Lhr)-positive cells in the ovarian stroma of aged mice, noting that these endocrine cells may produce excessive androgens associated with stroma fibrosis and inhibit FSH-stimulated secondary follicle development [85]. This plateau is probably because of increased luteinizing hormone (LH) stimulation on theca-interstitial cells as well as the increase in Lhr-positive cells [86]. Androgens are well known to play an essential role in early follicle development (primordial follicle activation and preantral follicle growth), serving as a substrate for estrogen, and fine-tuning the extracellular matrix and vasculature of the ovarian stroma [87]. The coculture of small follicles with stromal cells containing thecal cells and macrophages substantially promotes follicle growth and survival compared with follicles alone, probably through the mechanism of androgen [67]. In this respect, the results indicate that androgens may have a pleiotropic effect during the life of women, but more specific investigation is required to tease out how their actions change with age and what the consequences are.

The function of theca-interstitial cells is regulated by multiple factors, including estrogen, insulin signaling, and the circadian clock, etc., which could be the reason for the age-related dysfunction of these cells; however, direct studies about their age-related changes are lacking [73, 81]. Ethun et al. studied the relationship between theca cellular function and reproductive aging in macaques, reporting that decreased steroidogenesis of theca-interstitial cells is accompanied by a lowered follicle number with aging [74]. Estrogens promote the production of androgens in the stromal cells of goats, suggesting age-related weakening of the paracrine feedback loop between follicles and theca-interstitial cells that may lurk in the ovaries [75]. Deletion of the brain and muscle Arnt-like protein-1 (*Bmal1*) locus, a key factor controlling circadian rhythm, in ovarian theca cells in mice, leads to altered luteinizing hormone/choriogonadotropin receptor (*Lhcgr*) expression, loss of phasic sensitivity of LH, and impaired reproduction [88]. Because circadian desynchrony progresses with aging, a similar mechanism in aging women is implied [76, 89]. Ford J.H. proposed the decreased production of dehydroepiandrosterone (DHEA) and peroxisome proliferator-activated receptor alpha (PPARα) in aged theca cells. This led to follicle loss and oocyte apoptosis, which was implicated by the age-related decline in their downstream intermediates, i.e., androstenedione and ceramide [77, 78, 90]. The main senescence-associated marker, senescence-associated beta-galactosidase (SA-β-gal), is present in theca-interstitial cells rather than follicular cells, indicating that these cells may exert a pro-aging function via cellular senescence, i.e., through inflammation and tissue remodeling [79]. Collectively, gaps in knowledge remain regarding the elucidation of the role of LH receptor-positive cells in aged ovaries, and more age-related changes in the theca-interstitial cells.

#### Immune cells

A range of immune cells, including adaptive lymphocytes (i.e., T and B cells), monocytes and macrophages, natural killer (NK) cells, dendritic cells, and eosinophils, are found in ovarian tissues [29]. Generally, immune cells support ovarian physiology through phagocytosis, antigen presentation, the inflammatory secretome, and extracellular matrix remodeling, in which dysfunction may cause blunted immunosurveillance, hyperactive stress, or persistent inflammation. Matthew et al. recently demonstrated the key role of the immune system and senescent immune cells in organic damage and organism aging [80]. Nevertheless, these mechanisms in ovary require further investigation.

The innate immune response plays an essential role in follicular cycles, specifically in ovulation and corpus luteum regression. An early investigation suggested the role of mast cells in rat ovarian activity based on their distribution during the estrous cycle; however, there was no further study to elucidate the specific effect [91]. A recent investigation in mice showed increased CD4+ T cells, B cells, macrophages, and NK cells and decreased CD8+ T cells in aging ovaries, suggesting their relationship with follicle depletion [92]. Recent bioinformatics studies comparing the RNA sequencing data of young and aged mouse ovaries from the Gene Expression Omnibus (GEO) database also found increased expression of hallmarks of plasma cells and naïve CD4 T cells in aging mouse ovaries, implying their roles in ovarian aging [34]. Autoimmunity has been widely found in premature ovarian failure (POF), and this is accompanied by increases in CD19+CD5+ B cells and CD4(+) Th1 T cells and decreases in NK cells [35, 36]. Treg cells are essential for the maintenance of ovarian function, and their deficiency is the main cause of autoimmune ovarian disease [33]. Nevertheless, the investigation of immune cells in the ovary is still inadequate, and all the above results are either observational or bioinformatic inferences. Further studies need robust mechanical methods such as transgenic mice or targeted ablation to explore what their roles are during aging.

To date, macrophages (Mφ) have been the most explored immune cells in the ovary, and they are known as the most abundant immune cells in the ovary and a prominent hallmark of inflamm-aging [93]. According to the results of a recent single-cell sequencing study in humans, cells of the monocyte–macrophage system are the most predominant types in ovarian stroma following follicle cells [94]. Generally, according to their specific functions, macrophages are classified into two types. M1-like macrophages promote acute inflammation in the early stage of the immune response, whereas M2-like macrophages remodel tissue and resolve inflammation in the late stage. Macrophages have been found to promote the survival and growth of early follicle development [67]. Ono et al. demonstrated that M1-like macrophages played an indispensable role in the growth, vascularization, and estrogen production of follicles in mice through pericyte recruitment and granulosa cell proliferation [25]. Additionally, macrophages are also involved in proinflammation and matrix breakdown during ovulation [93]. During the luteal phase of many species, macrophages maintain the integrity of the vasculature and promote progesterone synthesis [26]. However, M1-like macrophages play a central role in interacting with luteal cells to regulate luteolysis [95–97]. In atretic follicles, endothelial cells recruit macrophages through interleukin-33 (IL-33) for the phagocytosis of apoptotic follicle cells, and a large amount of waste accumulates in the ovaries of IL-33-deficient mice [31]. In this current research perspective, macrophages are closely associated with ovarian physiology throughout the follicle cycle.

Previous studies have documented a few age-related changes in ovarian macrophages, including increased recruitment of monocyte-derived macrophages from circulation, decreased quantity of resident ovarian macrophages [92]. Over the course of aging, the total population of macrophages in mouse ovaries substantially declines and there is a shift from M1 to M2 polarization. Moreover, these macrophages become predisposed to replacement by monocyte-derived macrophage lines, and M2 macrophages are known to be closely associated with age-related chronic inflammation and fibrosis [29]. Additionally, stromal macrophages are overloaded with excessive nonheme ferric and ferrous iron in aged mouse ovaries, which may be due to oxidative stress [30]. Moreover, macrophage-derived multinucleated giant cells (MNGCs) are uniquely present in aged ovaries [98]. They are interpreted as a specific cell type that compensates for macrophages in response to aging-accumulated cell debris or waste from follicle atresia and luteolysis over the course of repeated ovulatory cycles [31]. Furthermore, MNGC has also been viewed as a hyperactive and fused form of macrophages caused by excessive accumulation of hemosiderin and lipofuscin [99, 100]. Blunted immune clearance leads to the accumulation of excessive lipofuscin or other metabolites, and this accumulation can augment oxidative stress and inflammation [101]. The results of studies of conditional knockout mouse models have indicated that the deficiency may contribute to abnormal folliculogenesis and corpus luteum formation through defective vasculature [25, 102]. Above all, more mechanical studies on the age-related alterations of macrophages in ovaries, such as M2 polarization and formation of MNGCs, are merited, and how these changes are associated with ovarian dysfunction needs further clarification.

#### Blood and lymphatic vasculature

The vasculature system provides a supportive architecture for follicles by delivering hormones, circulatory factors, oxygen, and precursors for metabolism and removing metabolic wastes [50]. The system prominently impacts the selection of dominant follicles and luteal hormone secretion. Vascular endothelial growth factor (VEGF) is the most important angiogenic factor during perifollicular vascularization, along with its receptor VEGFR-1/2 [103, 104]. Once antrum starts forming, angiogenesis occurs, and newly formed capillaries penetrate the thecal layers in response to hypoxia in the granulosa layers of preovulatory follicles [81]. After the LH surge and despite increased vascular support and permeability, transient and relative hypoxia caused by increased O2-binding hemoglobin induces the activation of hypoxia-inducible factor (HIF-1α)/VEGF/VEGFR signaling, thereby leading to extra vascularization. Hypoxia persists until early luteal formation because of the fast growth of the corpus luteum as well as vasculature degradation caused by ovulation [105, 106].

Wagner et al. identified six main types of cells in human ovaries with a single-cell analysis, among which perivascular cells (~10%) and endothelial cells (~5%) were most commonly present in the cortical stroma [107]. The results of another single-cell analysis study of human ovaries reported endothelial cells and smooth muscular cells constitute a significant part of the inner cortical stroma [94]. Endothelial cells and perivascular cells, mainly pericytes, play a crucial role in postovulatory vascularization. They serve as the initiators of angiogenesis in response to hypoxia, penetrating the hypoxic granulosa layer inside the follicles and aiding in subsequent capillary outgrowth. In later stages, pericytes are recruited for the maturation and stabilization of newly formed vessels [37, 38, 44]. In the condition of organic aging or age-related diseases, vascular cells undergo many unfavorable alterations. Pericytes differentiate into fibroblasts, causing fibrosis in the kidney, joints, etc. [41, 47, 108]. Additionally, they undergo apoptosis and detach from blood vessels, which is linked with neurodegeneration and diabetes [39, 40]. Endothelial cells manifest blunted regeneration, decreased endothelial nitric oxide synthase (NOS)-NO activity, and compromised relaxation, leading to vasospasm and hypoperfusion [43]. Additionally, microenvironmental stimuli, such as AGEs and ROS, will also participate in disrupting endothelial alignments, migration, VEGF responsiveness and their interaction with the ECM and pericytes [109, 110]. Moreover, the suboptimal angiocrine of aged endothelial cells has also been found to promote postovulatory inflammation and fibrosis [49]. In view of all the above, it is evident that blood vessel cells are essential for maintaining normal folliculogenesis, and there are multiple changes associated with aging. However, more detailed studies are warranted to validate these changes and clarify their specific roles in aging ovaries.

Aside from vascular cells, the microvascular network also manifests an age-related alteration in ovary. Ovaries experience continuous cyclic and highly controlled remodeling of vascular networks that accompanies folliculogenesis and luteinization. Consistent with multiple organs such as the kidneys, lungs, thymus, and heart, the ovaries show decreased vascularization and blood flow with aging, as demonstrated by three-dimensional (3D) power Doppler ultrasonography [51–53]. The volume density of blood vessels in the superficial cortex of normally cycling ovaries significantly increases after the age of 30, which is related to the accelerated depletion of primordial follicle reserves during the same period [54]. Consistently, hypoxia- and VEGF-induced angiogenesis in the perifollicular area also increase during ages 38–46 [111]. Small, avascular follicles mostly rely on the diffusion of oxygen and nutrients from nearby stromal blood vessels. Physiologically, the poor prepubertal ovarian vasculature is known to be associated with maintaining the dormancy of the primordial follicle pool and inhibiting primary follicle growth [27, 112]. These results indicate that the acceleration of primordial follicle activation in middle age might be linked with increased cortical vascularization. In aged ovaries, blood vessels undergo hyaline degeneration, sclerosis, and stenosis, resulting in insufficient blood supply and hypoxia [52]. This may be explained by the faster rate of vascular aging in medullary and deep cortical regions (specifically mid-sized arteries) and characterized by hyalinization, vessel-wall thickening, and lumen narrowing. This leads to blood flow decline and superficial cortical ischemia, culminating in terminal microvascularization [54].

In addition to blood vasculature alterations, lymphatic remodeling is ongoing during cyclic ovulation and is regulated by hormones such as FSH and estrogen [56]. Lymphangiogenesis in the ovary is predominantly mediated by VEGF-C/VEGF-D/VEGFR-3 signaling. Additionally, a disintegrin and metalloproteinase with thrombospondin type 1 motif-1 (ADAMTS-1), an extracellular metalloproteinase in the stromal microenvironment, is involved [113]. Based on its function in hormone recruitment, homeostasis of extravascular fluids, and immune cell transport, lymphangiogenesis has implications for several ovarian pathologies, such as PCOS, hyperstimulation syndrome, and malignancy. The study of blockage with VEGFR3 neutralization demonstrated the role of lymphangiogenesis in the development of estrogenic secondary follicles as well as in follicular fluid accumulation of early antral follicles [56, 57]. In aged lymphatic vasculature, capillary rarefaction is induced by fibrosis and lowered VEGF-C levels. The collecting vessels are dilated, less contractile, and more permeable, which is potentially associated with age-related pathologies such as inflammation and autoimmunity [55]. Additionally, lymphatic vascular diseases such as lymphedema exhibit a pronounced predominance in women, suggesting that age-related lymphatic dysfunction may play a role in ovarian aging [114].

#### Extracellular matrix (ECM)

In the ovaries, stromal cells express high levels of collagen, which provides structural support for follicles [81]. The ECM, together with sequestered growth factors and cytokines, plays a significant role in regulating intrinsic cellular functions and the cellular interactions between somatic components and germline cells (i.e., follicles and oogonial stem cells). The ECM is dynamically remodeled by enzymes such as matrix metalloproteinases (MMPs), tissue inhibitors of matrix metalloproteinases (TIMPs), and plasminogen activators, etc. [115]. The softening or degradation of the ECM produces fragments or releases sequestered molecules, mediating downstream signaling pathways in follicle cells [58, 63]. Additionally, the composition of the ECM determines the stiffness of the ovarian stroma, which affects primordial follicle activation and primary follicle growth [60]. Finally, ECM components, such as fibronectin and laminin, which contain integrin-binding sequences (most notably Arg-Gly-Asp (RGD)), directly regulate the proliferation of follicle cells as well as the differentiation of oogonial stem cells [65, 116].

In the ovarian stroma, age-associated increases in collagen (type I and type III) and hydroxyproline are associated with chronic inflammation and fibrosis in mice [61]. Similarly, an increased collagen content, together with a decreased hyaluronan (HA) content, is linked with suppressed ovulation, compromised oocyte competence, and reduced theca cell function and androgen production [62, 64]. The degradation of HA produces low-molecular-weight (LMW) hyaluronan, which is one of the best-characterized damage-associated molecular patterns (DAMPs) causing pathogen-free inflammation in the aged milieu. Consistently, Rowley et al. showed that in vitro exposure to LMW hyaluronan promotes the secretion of inflammatory cytokines and the recruitment of inflammatory cells; moreover, it impairs oocyte meiosis and granulosa cellular steroidogenesis [63].

### Microenvironmental molecules

**Fig. 3:**
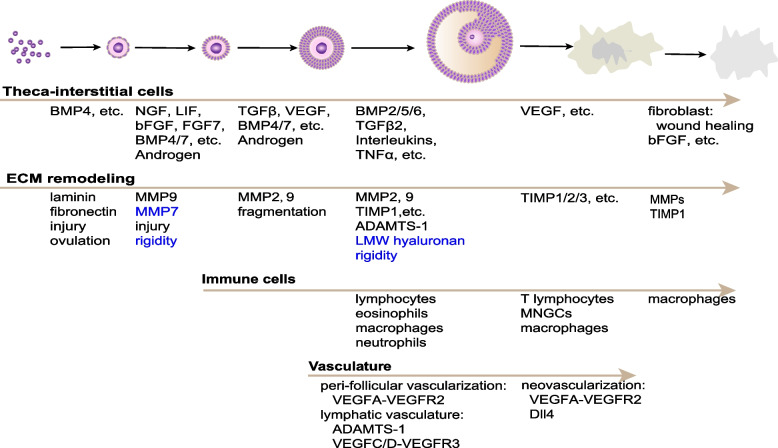
Stromal components associated with different stages of oogenesis, folliculogenesis, and corpus luteum. Black, up-regulation; blue, down-regulation

Factors such as oxidative stress, AGEs, inflammatory cytokines, and related fibrosis, have been implicated in organic aging and age-related pathological progression. Extensive research has demonstrated their involvement in ovarian pathology such as PCOS and premature ovarian failure (POI); however, the gap in knowledge pertaining to their roles in the stromal microenvironment during ovarian aging remains to be filled (Fig. 3).

#### Advanced glycation end-products (AGEs)

AGEs are generated by the Maillard reaction, polyol pathway, and lipid peroxidation, which can occur endogenously in vivo or exogenously through the daily diet [117]. AGEs are a family of long-lived molecules that are usually cross-linked with other long-lasting matrix proteins, i.e., collagen, which has been shown to increase in the ovarian stroma [62, 64, 118]. In reproductive-aged ovaries, AGE levels are 30 times higher because of protein glycation or cross-linking together with the compromised scavenging system [119]. In humans, AGEs also accumulate in ovaries over time by binding to their receptor RAGE [120]. AGEs have been implicated in the progression of PCOS and diabetes-associated ovarian dysfunction. Mechanistically, in granulosa cells, AGEs reduce glucose transporter type 4 (GLUT-4) expression and glucose uptake. This abnormally activates the extracellular signal-regulated kinase (ERK) 1/2 pathway and inhibits LH-stimulated steroidogenesis [121]. In mice, oocytes administered with the AGE precursor methylglyoxal exhibit abnormal meiotic spindles and aneuploidy [122, 123]. AGE accumulation increases lysyl oxidase activity, leading to abnormal collagen cross-linking and excessive ECM deposition, which promotes stiffness and microvascular sclerosis [124]. RAGE is broadly expressed among immune cells such as monocytes and macrophages; moreover, increased RAGE expression is associated with decreased immune clearance and the accumulation of AGEs in turn [120, 125]. AGEs can also regulate macrophage polarization and infiltration, impair endothelial alignment, and cause pericyte loss [109, 126, 127]. Moreover, AGEs interact with the receptor RAGE, leading to the activation of the downstream signaling pathways of NF-κB and NADPH oxidases (NOXs). This promotes inflammation and oxidative stress in the microenvironment [128]. 

#### Reactive oxidative species (ROS)

Oxidative species are the most abundant and inevitable byproduct of cell metabolism. Studies have reported that one of the main reasons for ovarian aging should be the decreased antioxidant ability of ovarian cells, leading to the accumulation of ROS [129, 130]. Macromolecules such as proteins, lipids, and nucleic acids can all be targeted. The influence of oxidative stress on follicle cells, including granulosa cells and oocytes, has been well established elsewhere [100, 131]. In the ovarian stroma, a moderate level of ROS can stimulate the proliferation of theca-interstitial cells, but a high-level of ROS leads to suppression [132]. Paradoxically, excessive oxidative stress has been implicated in stromal hyperplasia and androgen overproduction in PCOS [133]. Oxidative stress also inhibits the proliferation and migration of fibroblasts, which is a crucial modulator of ECM reconstitution and wound healing [134]. Macrophages are more vulnerable to oxidative stress in aged mice than in younger mice [101, 135]. ROS disrupt the maturation, differentiation, polarization, and phagocytosis of macrophages [136]. Lipofuscin accumulates in macrophages and promotes ROS production [101]. Moreover, various aspects of the vasculature have also been shown to be affected by ROS, including endothelial development, pericyte coverage, endothelial–pericyte interaction, adhesion of the endothelium to the ECM, VEGF-A response, and endothelium-related vasodilation [110, 137]. However, detailed investigations are still needed to clarify the roles of ROS in the aging ovarian microvasculature.

#### Inflammatory cytokines

Chronic, low-grade inflammation usually occurs with advanced aging as a result of accumulated damage to macromolecules, uncontrolled stress responses, and dampened innate immunity [138]. Inflammatory cytokines, such as interleukin (IL)-6, IL-1 and tumor necrosis factor (TNF)-α, have been implicated in follicle development in a complicated manner. In a mouse model, the deletion of IL-1α led to improved reproductive performance, which was associated with elevated AMH levels, increased ovarian response, and resistance to apoptosis [139]. Consistently, in TNFα-/-mice, ovarian performance was improved by TNFα knockout through the mechanism of decreased oocyte activation and cell apoptosis [140]. Inflammasome-associated adaptor, apoptosis-associated speck-like protein containing a caspase activation and recruitment domain (ASC), Nod-like receptor family, a pyrin domain containing (NLRP) 3, and IL-18 were increased in the aging ovaries of mice [92]. Additionally, inflammasome-induced low-grade chronic inflammation was demonstrated to be involved in follicle reserve diminishment using *Asc*−/− and *Nlrp3*−/− mouse models [141, 142]. Nevertheless, there is a dearth of studies on the effect of chronic, low-grade inflammation on the cellular compartments of the ovarian stroma. Our group has identified a series of proinflammatory cytokines (chemokine ligand (CCL)9, CCL11, CCL5, and IL-6, etc.) secreted by stromal cells from ovaries of reproductively aged mice and found their inhibitory effect on follicle development [79]. Moreover, the chronic, low-grade inflammation in aged tissue leads to fibrosis through the stimulation of ECM deposition and abnormal remodeling, which indicates its role in altering the peri-follicular microenvironment with aging, however, further research is warranted [61, 143].

## Fibrosis

Fibrosis is a significant characteristic of the stroma of multiple organs that influences their function. Age-associated fibrosis is known to occur in the ovaries of mice and humans [61, 144]. Cyclic ovulation, viewed as a repeated process of inflammation and wound healing, acts as a persistent irritant, resulting in fibrosis [145, 146]. In aged ovaries, fibrosis in the stroma has been found to be related to impaired ovulation and postovulatory tissue remodeling [147, 148]. MMP and TIMP coordinate with each other to balance the synthesis and degradation of ECM; however, they can be disrupted by multiple profibrotic cytokines. An increased fibroinflammatory cytokine profile in the ovary has been found to be inversely correlated with the reproductive performance, and TGF-β3 was specifically linked with fibrosis of the ovarian stroma and vasculature [149]. Collagens are strongly associated with the extent of fibrosis. Depletion of collagen by enzymes or drugs (pirfenidone and BGP-15) was shown to eliminate fibrosis and, moreover, to rejuvenate ovarian structure and extend the reproductive life of aged mice [62, 150].

Umehara et al. found two heterogeneous interstitial cell types, i.e., Lhr+ endocrine cells and actin-rich fibrotic cells in the ovarian stroma of a 6-month-old mutant mouse model of accelerated aging (the granulosa cell-specific Nrg1 knockout mice (gcNrg1KO)) as well as 12-month-old WT mice. They proposed that stromal fibrosis in the aging ovary is caused by elevated LH secretion via the stimulation of these two cell types. With gonadotrophin-releasing hormone (GnRH) -antagonist treatment, aberrant endocrine cells and fibrotic cells were removed, and ovarian function was restored [151]. Additionally, McCloskey et al. studied fibrosis in aging ovaries of humans and mice and discovered that it was associated with the activation of dipeptidyl peptidase 4 (DPP4) + α-smooth muscle actin (α-SMA) + fibroblasts, which are a profibrotic subset of fibroblasts [144]. DPP4 inhibitors have also been demonstrated to be able to alleviate age-related renal fibrosis, implying that DPP4 is a potential therapeutic target in the ovary [152]. Using scRNA-seq, Landry et al. identified a type of fibroblast, secreting senescence-associated secretory phenotype (SASP), in age-associated ovarian fibrosis, implying cellular senescence among fibroblasts [153]. In addition to fibroblasts, infiltration of certain immune cells such as M2 macrophages and CD8+ T cells, and an increased CD206+/CD68+ cell ratio is also implicated in fibrotic ovaries [144]. As a hallmark of inflamm-aging, M2 polarization of macrophages is known to be increased in the old ovaries of mice and promotes collagen deposition. Metformin, a well-known antiaging drug, was shown to prevent fibrosis in mouse ovaries by suppressing CD8+ T-cell infiltration and the CD206+/CD68+ cell ratio, as well as clearing senescent fibroblasts [144]. However, the change in CD8+ T-cell percentage in aged ovaries is ambiguous and this needs more exploration [92]. Nevertheless, there is insufficient evidence in favor of the role of these cells in age-related fibrosis and ovarian dysfunction, and more explorations are warranted to identify the mechanism of age-related fibrosis in the ovaries and the potential of antifibrotic drugs in ovarian aging.

Fibrosis can cause stiffness and increase the rigidity of tissues. In the ovary, the biomechanics theory has been proposed to explore the mechanism of physical rigidity on follicles, i.e., the dormancy of immature oocytes through nuclear rotation and FOXO3a inhibition, the inhibition of early follicle growth by actin polymerization, and the Hippo pathway [60, 154]. In an in vitro culture system, rigidity significantly influences the growth, antral formation, and oocyte quality of secondary follicles [155, 156]. Follicle development and hormone secretion can be restored in aged mice through surgical cutting, possibly due to the release of mechanical stress, resolution of fibrosis, or reconstruction of the injury–repair system [154, 157]. Consistently, Bouzin et al. observed that increased rigidity in human ovaries at both prepuberty and postmenopause is probably associated with the inhibition of follicle activation [27]. Notwithstanding, more detailed work is needed to delineate the change in rigidity during reproductive life years and the biomechanical characteristics of accelerated follicle activation at mid-age.

## Future perspectives on microenvironment-based strategies

### Stem cell-based therapy

**Fig. 4:**
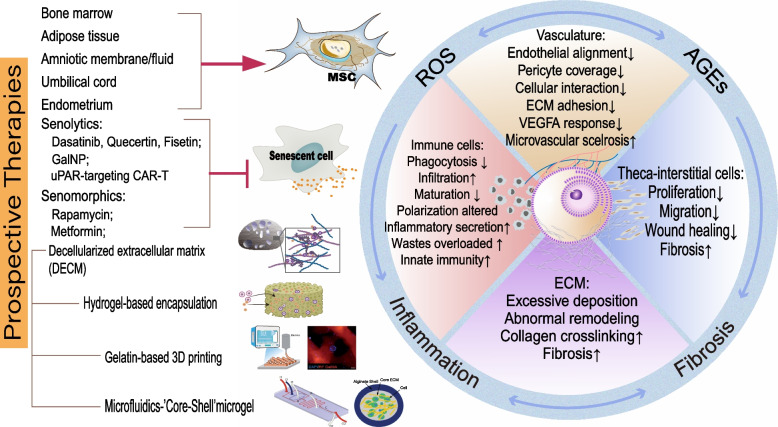
Microenvironmental factors (ROS, AGEs, inflammation, and fibrosis) entwined with each other, leading to age-related alterations in stomal components. (Photographs from Wu et al. [167] and Agarwal et al. [168] with required copy right permission)

Stem cell-based therapy holds considerable promise for the treatment of infertility. Preclinical studies have shown that ovarian failure can be recovered by the transplantation of mesenchymal stem cells (MSCs) from different sources, such as bone marrow, adipose tissue, amnion, umbilical cord, menstrual blood, etc. [158–162] (see Fig. 4). Compared with other stem cells (e.g., induced pluripotent stem cells (iPSCs) and embryonic stem cells (ESCs)), autologous mesenchymal stem cells have advantages in clinical application owing to their abundance, high accessibility, low immunogenicity, and stability. In a recent study, rat MSCs were observed to spontaneously translocate to the interstitial rather than intrafollicular region, suggesting the essential role of stromal compartment mediating the effect of MSCs [163]. Principally, MSCs improve the local environment of follicles through ECM remodeling, lymphangiogenesis, immune cell recruitment, and inflammation modulation. Human menstrual blood-derived stromal cells (MenSCs) have been shown to restore ovarian function after chemotherapy through the ECM-dependent FAK/AKT pathway and maintain the homeostasis of the microenvironment [162]. In a rat model of ovarian dysfunction with ovariectomy, Cho et al. showed the restorative effect of placental-derived mesenchymal stem cells by increasing angiogenesis and vascular remodeling via the VEGF signaling pathway [159]. The administration of human umbilical cord mesenchymal stem cells has been revealed to inhibit inflammation and fibrosis in ovarian tissue by downregulating the expression of TNF-α, IL1β, IFN-γ, and CTGF [164]. Based on their multipotency, MSCs may also aid in ovarian recovery by differentiating into stromal cells as a substitute for their senescent or apoptotic counterparts. Moreover, the transfer of micro-RNAs (miRNAs), exosomes, and mitochondria from MSCs to neighboring cells has been reported in many health conditions and diseases [165, 166]. Alternatively, an elaborate combination of beneficial stem cell-secreted factors may be used as a therapeutic molecular panel in the future to treat ovarian dysfunction.

### Senotherapy

Cellular senescence is characterized by permanent and irreversible cell cycle arrest while being antiapoptotic and metabolically active. Senescent cells persist in tissue for years, waiting to be cleared by immune system. These cells are usually identified by their enlarged, flattened morphology and molecular hallmarks, i.e., β-galactosidase (SA-β-gal), gamma H2A histone family member X (γ-H2AX), heterochromatic foci (senescence-associated heterochromatic foci, SAHF), and specific secretome (SASP) [169]. Age-related deposition of certain senescent markers, i.e., lipofuscin aggresomes, p21^WAF1^, and p16^INK4A^, has been found in mouse ovaries and is negatively correlated with the size of the primordial follicle pool [170]. However, the mechanism of action of cellular senescence in ovarian pathophysiology remains largely unexplored. In our previous study, SA-β-gal activity was observed to be increased in aged mouse ovaries, whereas the accumulation of lipofuscin and SA-β-gal foci was specifically observed in stromal cells [79]. Landry et al. have also identified a type of SASP-associated Cd74Hi fibroblast in aged mice and found that these cells may evade immune clearance and persist in aging ovaries [153]. In follicles, granulosa cells undergo either proliferation or apoptosis rather than being in long-term cell cycle arrest. Oocytes stay in quiescence and do not undergo replication. As the consequence of limited replication and being nonproliferative and antiapoptotic, cellular senescence is more likely to occur in the stromal cells of the ovary. Consistently, existing studies have only demonstrated that granulosa cells are induced to senescence in vitro or ex vivo, while no observations of in vivo senescence of granulosa cells have been reported [171–173].

To date, senotherapy, i.e., therapies targeting cellular senescence and its nonautonomous effects, has been developed and validated in a wide range of organs [174]. It is a potential strategy to alter the microenvironment of aging ovaries without perturbing the follicles (see Fig. 4). Senotherapy is classified into two types: (1) Senolytics, which eliminate senescent cells by targeting antiapoptotic pathways, i.e., ABT263 and dasatinib, which inhibit B-cell lymphoma protein-2 (Bcl-2) family proteins [175, 176]; heat shock protein 90 (HSP90) inhibitors, which destabilize phosphorylated AKT [177]; and the FOXO4 peptide, which dissociates FOXO4-p53 and releases nuclear p53 [178]. (2) Senomorphics, which mitigate the cell-extrinsic effects of senescent cells, primarily by targeting SASP or its upstream mechanisms, such as directly neutralizing SASP factors or antagonizing their receptors (i.e., siltuximab, tocilizumab), modulating NF-κB transcription (metformin, kaempferol), and many others [179, 180]. Many senotherapeutic candidates, such as rapamycin and metformin, have been shown to be able to restore the ovarian function with aging [181, 182]. Senolytics induce the ablation of senescent cells in mice enabling the rescue of a variety of aging-related symptoms, improving metabolism and prolonging lifespans [183–185]. In the context of reproductive systems, the senolytic regimen of dasatinib plus quercetin (D+Q) has been shown to ameliorate dysfunction and fibrosis in uterus of aged mice [186]. Our group previously reported that the senotherapeutic compound D+Q attenuated stromal fibrosis and protected the ovarian function from the cisplatin treatment through the removal of senescent cells [187]. Innovative techniques such as nanoparticles and immunotargets have recently been applied into the field of senotherapy. Galactose-conjugated nanoparticles (GalNPs), called senoprobes, have been developed as a drug delivery system by encapsulation with galacto-oligosaccharides to target senescent cells. With the sensitization of the high activity of β-galactosidase in lysosomes, the senoprobe is degraded and releases cytotoxic drugs preferentially in senescent cells. This gal-encapsulated biomaterial has been shown to reduce chemotherapy-induced cellular senescence and lung fibrosis in mice [188]. Based on the recognition of the surface protein urokinase plasminogen activator receptor (uPAR), Corina Amor et al. developed the senolytic chimeric antigen receptor (CAR)-T cells to target senescent cells. Similarly, they have also shown that uPAR- specific CAR-T cells could ablate senescent cells and alleviate the adverse effects of senescence-inducing chemotherapy [189]. The results of these studies have established the therapeutic potential of senolytic techniques in the ovary, but further investigation is needed.

### Tissue engineering

Currently, ovarian tissue and follicle cryopreservation and in vitro maturation are considered future research hotspots because of the increasing trend of delayed pregnancy and high cancer incidence among younger generations. Tissue engineering has been developed to restore the normal microenvironment of cryopreserved follicles and/or tissues (see Fig. 4). As mentioned above, the ECM provides a mechanical scaffold for follicle embedding and resumption. In classic 3D-hydrogel-based culture systems, the development of follicles is largely dependent on the composition of the hydrogel, alginate, incorporated growth factors, and ECM peptides, all of which must be in a precise state to achieve ideal mechanics [190]. New techniques, such as tethering matrix proteins and/or affinity bounding of growth factors, have also been introduced into the systems to produce a more suitable artificial scaffold and mimic the native microenvironment [58, 191]. The modification of hydrogels with synthetic biomaterials, such as ﻿poly (ethylene glycol) (PEG) has been applicated in the field of artificial ovaries. PEG-based hydrogels crosslinked with MMP-sensitive peptides have been shown to improve the microenvironment of follicles and the survival rate [192]. A supramolecular hydrogel which coated with a receptor tyrosine kinase (RTK) inhibitor has been developed to delay the ovarian aging by inhibiting the RTK-mTOR pathway [193]. An alternative to synthetic scaffolds is decellularization, which provides a natural and acellular ECM scaffold of the whole organs or tissues. Studies in rodents have shown that decellularized ovaries from mammals such as bovines and porcines could support the survival and maturation of follicles [194–196]. Laronda et al. reconstructed ovaries with decellularized ovarian scaffolds from bovines or humans and ovarian cells from mice. They demonstrated that the steroidogenesis had been recovered in vitro and that puberty in ovariectomized mice had been initiated after the transplantation [197]. Another transplantation study with human ovarian scaffolds and rat ovarian cells have also revealed the feasibility of decellularization in the reconstruction of the artificial ovaries [198]. However, there are still some safety concerns about the decellularization protocol, i.e., the usage of detergents. Moreover, recent advancements in biomaterials and techniques have shown their potential for application in ovarian aging. Gelatin-based 3D printing of ovarian scaffolding has been shown to successfully seed follicles and restore the ovarian function. Additionally, the ovarian graft has achieved live birth after implantation in sterilized mice [199]. Furthermore, our group compared the printability of different biomaterials for follicle growth and found that gelatin-methacryloyl (GelMA) was able to build a more appropriate microenvironment for follicle maturation [167]. To mimic the heterogenetic mechanics between the cortex and medulla, core–shell microgels were developed by incorporating different hydrogels (alginate and collagen) to encapsulate pre-antral follicles. This biomimetic ovarian microtissue has been shown to be able to maintain the preantral development and ovulation of secondary follicles [168, 200]. Moreover, the recent studies have shown that follicles and oogonia can be regenerated from the iPSCs of mice and humans in vitro [201, 202]. These achievements enable the construction of a *de novo* artificial ovary based on the biomimetic scaffolding from tissue engineering, and follicles as well as stromal cells derived from patient iPSCs. However, more in-depth investigations are needed as these approaches are still in their infancy. Additionally, many differences exist between humans and other mammals.

## Conclusion

Ovarian dysfunction leads to the initiation and progression of many age-related pathophysiological conditions, such as osteoporosis, diabetes, cardiovascular diseases, and neurodegenerative diseases, which negatively impact a woman’s quality of life [203]. Ovarian aging is characterized by declines in the quantity and quality of follicles. Additionally, newly identified oogonial stem cells have also been proposed to be a potential substitute for follicles to reverse ovarian failure. Here, we provided a detailed description of the age-related alterations of the stromal microenvironment and their mechanism of action leading to ovarian failure. Furthermore, based on the current technological platforms, i.e., stem cell-based regeneration, tissue engineering, and cell targeted therapy, several new and emerging strategies will be developed to cure age-related infertility and ovarian senescence. However, uncertainties still exist, such as senescence of follicle cells, the off-target toxicity of senotherapy and the safety of biomaterials and stem cells. Thus, achieving further advances in all these areas is dependent on more sensitive detection methods and prudent investigations in the future.

## Data Availability

Not applicable.
